# Risk factor of neonatal mortality in Ethiopia: multilevel analysis of 2016 Demographic and Health Survey

**DOI:** 10.1186/s41182-021-00303-5

**Published:** 2021-02-04

**Authors:** Setegn Muche Fenta, Hailegebrael Birhan Biresaw, Kenaw Derebe Fentaw

**Affiliations:** Department of Statistics, Faculty of Natural and Computational Sciences, Debre Tabor University, Debra Tabor, Ethiopia

**Keywords:** Neonatal mortality, Multilevel analysis, Ethiopia

## Abstract

**Background:**

In sub-Saharan African countries, neonatal mortality rates remain unacceptably high. Ethiopia is one of the countries in Sub-Saharan Africa with the highest death rates of newborn children. Therefore, this study aimed to identify the risk factors associated with neonatal mortality in Ethiopia at the individual and community level.

**Methods:**

The 2016 Ethiopian Demographic and Health Survey data was accessed and used for the analysis. A total of 2449 newborn children were included in the analysis. The multilevel logistic regression model was used to identify the significant factor of neonatal mortality. Adjusted odds ratio with a 95% confidence interval and *p*-value < 0.05 in the multilevel model was reported.

**Results:**

A total of 2449 newborn children were included in this study. Multiple birth type (AOR = 3.18; 95% CI 2.78, 3.63), birth order of ≥ 5 (AOR = 2.15; 95% CI 1.75, 2.64), pre-term birth (AOR = 5.97; 95% CI 4.96, 7.20), no antenatal care (ANC) visit during pregnancy (AOR = 2.33; 95% CI 2.09, 2.61), not received TT injection during pregnancy (AOR = 2.28; 95% CI 1.92, 2.71), delivered at home (AOR = 1.99; 95% CI 1.48, 2.69), less than 24 months of preceding birth interval (AOR = 1.51; 95% CI 1.35,1.68), smaller birth size (AOR = 1.58; 95% CI 1.46, 1.71), never breastfeeding (AOR = 2.43; 95% CI 2.17, 2.72), poor wealth index (AOR = 1.29; 95% CI 1.17,1.41), non-educated mothers (AOR = 1.58; 95% CI 1.46, 1.71), non-educated fathers (AOR = 1.32; 95% CI 1.12, 1.54), rural residence (AOR = 2.71; 95% CI 2.23, 3.29), unprotected water source (AOR = 1.35; 95% CI 1.16, 1.58), and have no latrine facility (AOR = 1.78; 95% CI 1.50, 2.12) were associated with a higher risk of neonatal mortality. Neonates living in Amhara, Oromia, Somali, Harari, and Dire Dawa had a higher risk of neonatal mortality compared to Tigray. Moreover, the random effects result showed that about 85.57% of the variation in neonatal mortality was explained by individual- and community-level factors.

**Conclusions:**

The findings suggest that attention be paid to education-based programs for mothers that would highlight the benefits of delivery care services, such as ANC visits, TT injections, and facility births. Meanwhile, public health initiatives should focus on expanding access to quality sanitation facilities, especially for latrines and drinking water that could improve neonatal health at the community-level as a whole.

## Background

An integral part of reducing under-five mortality is the reduction of neonatal mortality. Neonatal mortality is an important indicator of children’s well-being and health [1]. It is defined as death among live births during the first 28 completed days of life. Children who die during the first 28 days of birth suffer from complications and illnesses that are related to a lack of adequate birth care or professional care and treatment immediately after birth and during the first days of life [2, 3]. Country leadership has been critical to strengthening engagement, action, and partner harmonization efforts toward the implementation of the Every Newborn Action Plan which targets the reduction of the neonatal mortality rate to 12 or less per 1000 live births and stillbirths to 12 or less per 1000 births in all countries by 2030 [1, 3]. Worldwide, 2.4 million children died in the first months of life in 2019. There are around 7000 newborn deaths every day. About one-third of all neonatal death occurs on the first day after birth, and almost three-quarter occur in the first week of life. Most neonatal death results from preterm birth, intrapartum-related complications (birth asphyxia or lack of breathing at birth), infections, and birth defects [4, 5].

In Sub-Saharan Africa and South Asia, neonatal mortality was the highest, with neonatal mortality rates reported at 27 and 25 deaths per 1000 live births in 2019, respectively. A child born in Sub-Saharan Africa was 10 times more likely than a child born in a high-income country to die in the first month [6, 7]. The sustainable development goals had the target to end preventable death of newborns and children less than 5 years of age. The goal is for all countries aiming to reduce neonatal mortality to at least as low as 12 per 1000 live births [8].

In recent decades, Ethiopia has achieved tremendous progress in reducing neonatal mortality, but there is still a very high rate of neonatal mortality (29 deaths per 1000 live births). The country is among the 10 countries that account for 59% of neonatal deaths worldwide. Ethiopia is among the highest level of the child and neonatal morbidity and mortality rate compared to the sub-Saharan countries with a neonatal mortality rate of 29 per 1000 live births in 2016 [7, 9, 10]. Prior studies in Ethiopia have shown that neonatal mortality is one of the main health problems in the country [11–13].

Previous studies conducted in Ethiopia to investigate the risk factors of neonatal mortality were institutional-based [14, 15] and considering only individual-level factors [11–13]. However, neonatal mortality can be affected by community-level factors, such as the source of drinking water [16, 17], types of toilet facilities [18, 19], and cluster (enumeration area) [20, 21]. Moreover, the uses of a single-level logistic regression analysis approach to analyze data with a hierarchical structure (i.e., neonates nested within communities) violate the regression’s independence assumptions [22]. This study used multilevel logistic regression analysis to overcome these limitations and to further estimate the significant impact of individual- and community-level variables in Ethiopia. Therefore, this study aimed to identify the risk factors associated with neonatal mortality in Ethiopia at the individual and community level.

## Methods

### Study setting, data source, and study design

**Fig. 1:**
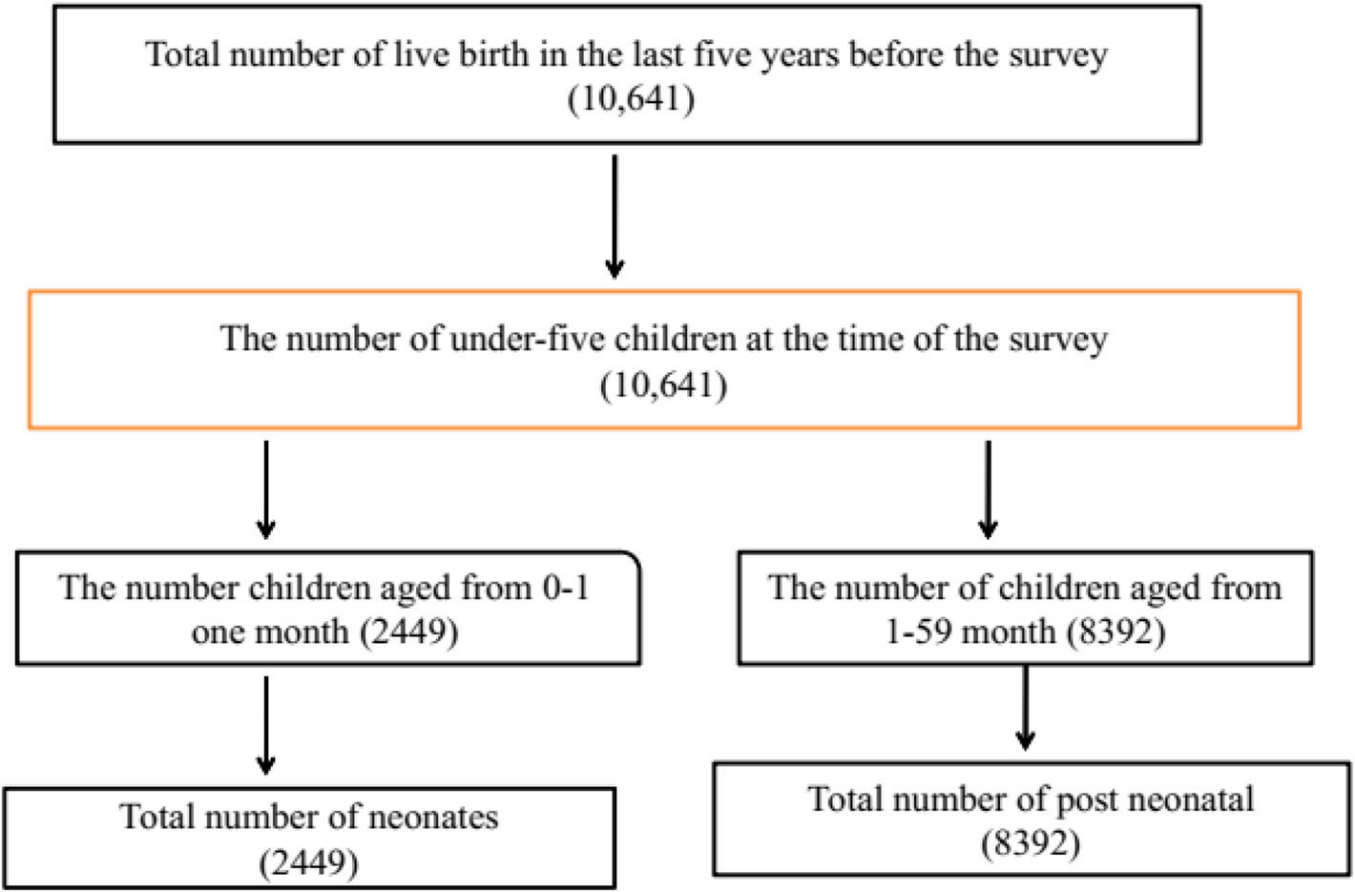
Total number of neonates included in the analysis in 2016 EDHS

This study was carried out in Ethiopia, and Ethiopia was the second-most populous country in Africa next to Nigeria and found in the horn of Africa [10]. The administrative structure of Ethiopia consists of nine regional states (Tigray, Afar, Amhara, Oromiya, Somali, Benishangul-Gumuz, Southern Nations Nationalities and People (SNNP), Gambela, and Harari) and two city administrations (Addis Ababa and Dire Dawa) [9]. A secondary data source from 2016 EDHS was used. This is the fourth national representative survey done at the country level. The main goal of this dataset was to provide up-to-date information about the key demographic and health indicators. The stratified multi-stage cluster sampling was used, and it was intended to be representative at the regional and national level in terms of appropriate demographic and health indicators. In the first stage, 645 clusters of enumeration areas (EAs) (202 urban and 443 rural) were identified using probability proportional to the size of EAs. In the second stage, random samples of 18,008 households were selected from all the identified EAs. Lastly, 16,650 households were successfully interviewed, yielding a response rate of 98%. The primary aim of 2016 EDHS was to provide up-to-date information about the key demographic and health indicators. Both men and women aged 15–59 years were interviewed. Data was also collected from mothers or caretakers of live-born infants in the 5 years preceding the date of the interview. A total of 2449 neonates were included (Fig. 1). The recorded data was accessed at www.measuredhs.com on request with the help of ICF International, Inc [9].

### Outcome variables

Neonatal mortality was the outcome variable for this study, which was defined as the death of a live birth before celebrating the first month of the day.

### Independent variables

The possible variables associated with neonatal mortality have been classified as individual- and community-level factors. These variables were selected on the basis of different works of literature [11–13, 18, 23]. The variable at the individual level was the age of mother, age at first birth, sex of household head, marital status, wealth index, mother and father educational status, occupational status, size of child at birth, number of living children, child sex, birth order, duration of pregnancy, preceding birth interval, birth type, place of delivery, number of antenatal care (ANC) visits, and number of tetanus toxoid (TT) injections during pregnancy. Place of residence, cluster (enumeration area), source of drinking water, availability of toilet facility, and region were the community-level variables.

### Data management and analysis

Data were extracted using the SPSS version 21 software and then exported to R version 3.5.3 statistical software for further analysis. Descriptive statistics including frequencies, percentages, and bar charts were performed to describe the study participants. A multilevel logistic regression model was used to identify factors associated with neonatal mortality. Four models were fitted for this multilevel analysis. The first model was developed without independent variables to assess the effect of community-level variation on neonatal mortality. Individual-level variables were incorporated in the second model. The community-level variable was included in the third model. Finally, in the fourth model, both individual- and community-level variables were included. The result of the fixed effect was reported in terms of adjusted odds ratio with a 95% CI. All variables with *p* values ≤ 0.05 have been considered statistically significant. The random effects measures the variation of neonatal mortality across clusters and expressed by ICC, median odds ratio (MOR), and proportional change in variance (PCV) [24]. Multicollinearity was tested using the generalized variance-inflation factor (GVIF) test, suggesting that there was no multicollinearity since all variables had GVIF< 5. Model comparison was done using deviance information criteria (DIC), Akaike’s Information Criterion (AIC), and Bayesian’s Information Criterion (BIC). The model with the smallest value of the information criterion was selected as the final model of the analysis [25].

### Ethical consideration

Publicly available EDHS 2016 data were used for this study. Informed consent was taken from each participant, and all identifiers were removed

## Result

### Sociodemographic characteristics of respondents

The number of respondents included in the study was 2449. Most (54.4%) of the respondents were in the 25–34 age group. The larger and the least proportion 370 (15.1%) and 115 (4.7%) of respondents were from Oromia regional state and Dire Dawa city of administration respectively. More than half (58.6%) of respondents were not formally educated; majorities (80.4%) of women were rural dwellers. Three-fourths (78.1%) of respondents were housewives, and 52.9% of the respondents were at low-income levels. More than two-thirds (68.4%) of women did not have a safe/protected source of drinking water, and 68.4% of women were married (Table 1).

**Table 1:** Socio-demographic characteristics of the respondents, EDHS 2016

Variable	Categories	Frequency	Percent
Mothers age	15–24	825	33.7
	25–34	1332	54.4
	35–49	292	11.9
Mothers educational status	No education	1436	58.6
	Primary	699	28.5
	Secondary and above	314	12.8
Family size	≤ 4	773	31.6
	> 4	1676	68.4
Wealth index	Poor	1295	52.9
	Middle	329	13.4
	Richer	825	33.7
Current marital status	Separated	129	5.3
	Married	2320	94.7
Mothers occupation	Housewife	1913	78.1
	Employed	536	21.9
Husbands’ educational status	No education	1050	42.9
	Primary	807	33.0
	Secondary and above	592	24.2
Sex of household head	Male	1950	79.6
	Female	499	20.4
Region	Tigray	255	10.4
	Afar	236	9.6
	Amhara	220	9.0
	Oromia	370	15.1
	Somali	361	14.7
	Benishangul	192	7.8
	SNNPR	289	11.8
	Gambela	146	6.0
	Harari	146	6.0
	Addis Ababa	119	4.9
	Dire Dawa	115	4.7
Source of drinking water	Protected	751	30.7
	Unprotected	1698	69.3
Improved toilet facility	Yes	1351	55.2
	No	1098	44.8
Residence	Urban	480	19.6
	Rural	1969	80.4

### Obstetric characteristics of respondents

The obstetric characteristics of respondents are summarized in Table 2. More than three-fourths (76.6%) of women’s age at first birth was greater than 16 years, and the majority of women (55.7%) gave birth to their child at home. About 33.6% of women did not have visits during pregnancy, two-thirds of children (65.3%) were receiving vaccination, and 46.3% of women did not receive tetanus injection during pregnancy. Only 2.9% of the children were never fed on their mother’s breast (Table 2).

**Table 2:** Obstetric characteristics of respondents, EDHS 2016

Variable	Categories	Frequency	Percent
Age of respondent at 1st birth	≤ 16	572	23.4
	> 16	1877	76.6
Number of living children	< 4	1407	57.5
	≥ 4	1042	42.5
Sex of child	Male	1220	49.8
	Female	1229	50.2
Place of delivery	Home	1363	55.7
	Health facility	1086	44.3
Birth order number	first order	524	21.4
	2–4	1079	44.1
	≤ 5	846	34.5
Type of birth	Single birth	2385	97.4
	Multiple birth	64	2.6
Size of child at birth	Larger than average	659	26.9
	Average	1020	41.6
	Smaller than average	770	31.4
Duration of breastfeeding	Ever breastfed, not currently breastfeeding	190	7.8
	Never breastfed	71	2.9
	Still breastfeeding	2188	89.3
Preceding birth interval	≤ 24	401	16.4
	> 24	2048	83.6
Number of TT injections during pregnancy	Not received	1134	46.3
	1–3	1155	47.2
	≥ 4	160	6.5
Vaccination of child	Yes	1600	65.3
	No	849	34.7
Number of ANC visits during pregnancy	No visit	822	33.6
	1–3	756	30.9
	≥ 3	871	35.5

### Determinant factors associated with neonatal death in Ethiopia

Table 3 summarizes the result of the two-level mixed-effect logistic regression model. The model selection result indicated that model IV was a better fit for the data as compared to other reduced models since it has the smallest AIC, BIC, and deviance statistics. The result of the full model revealed that sex of child, age at first birth, number of ANC visits, preceding birth interval, birth order, number of TT injection during pregnancy, type of birth, place of delivery, size of child at birth, duration of breastfeeding, mothers age, husbands educational status, maternal educational status, and wealth index were individual-level factors associated with neonatal death. In addition place of residence, region, source of drinking water, and type of toilet facility were community-level significant factors associated with neonatal death (Table 3).

**Table 3:** Multilevel logistic regression analysis for risk factors of neonatal death in Ethiopia, EDHS 2016 data

Variables	Model I AOR (95% CI)	Model II AOR (95% CI)	Model III AOR (95% CI)	Model IV AOR (95% CI)
**Individual-level factors**				
Sex of neonate				
Male		1		1
Female		0.59 (0.47,0.73)		0.59 (0.42, 0.83)*
Age of respondent at 1st birth				
≤ 16		1		1
> 16		0.71 (0.65,0.78)*		0.75 (0.59, 0.95)*
Number of ANC visits during pregnancy				
≥ 4		1		1
1–3		1.16 (1.05, 1.28)*		1.09 (1.01, 1.18)*
No visit		2.70 (2.37, 3.09)*		2.33 (2.09, 2.61)*
Duration of pregnancy				
Term		1		1
Pre-term		6.70 (5.57, 8.06)*		5.97 (4.96, 7.20)*
Preceding birth interval in month				
> 24		1		1
≤ 24		1.62 (1.45, 1.81)*		1.51 (1.35, 1.68)*
Birth order				
First order		1		1
2–4		1.16 (1.06, 1.28)*		1.51 (1.28, 1.79)*
≥ 5		1.93 (1.77, 2.10)*		2.15 (1.75, 2.64)*
Number of TT injections during pregnancy				
≥ 4		1		1
1–3		1.51 (1.28, 1.79)*		1.54 (1.30, 1.82)*
Not received		2.20 (1.86, 2.61)*		2.28 (1.92, 2.71)*
Type of birth				
Single birth		1		1
Multiple birth		4.60 (4.09, 5.17)		3.18 (2.78, 3.63)*
Place of delivery				
Health facility		1		1
Home		2.23 (1.66, 2.98)*		1.99 (1.48, 2.69)*
Size of child at birth				
Average		1		1
Smaller than average		1.92 (1.77, 2.09)*		1.58 (1.46, 1.71)*
Larger than average		1.15 (0.98, 1.34)		1.09 (0.88, 1.33)
Duration of breastfeeding				
Still breastfeeding		1		1
Ever breastfed, not currently breastfeeding		1.22 (1.01,1.47)*		1.12 (1.01, 1.24)*
Never breastfed		2.70 (2.37, 3.09)*		2.43 (2.17, 2.72)*
Mothers age				
15–24		1		1
25–34		1.10 (0.94, 1.29)		1.10 (0.85, 1.40)
35–49		2.12 (1.76, 2.55)*		2.03 (1.68, 2.46)*
Mothers’ education				
Secondary and above		1		1
Primary		1.35 (1.10, 1.65)*		1.21 (1.04, 1.40)*
No education		1.66 (1.35, 2.04)*		1.58 (1.46, 1.71)*
Wealth index				
Richer		1		1
Middle		1.39 (1.25, 1.55)*		1.21 (1.06, 1.37)*
Poor		1.52 (1.41, 1.64)*		1.29 (1.17, 1.41)*
Fathers’ education				
Secondary and above		1		1
Primary		1.28 (1.10, 1.50)*		1.19 (1.02, 1.39)*
No education		1.34 (1.14, 1.57)*		1.32 (1.12, 1.54)*
**Community-level factors**				
Residence				
Urban			1	1
Rural			2.06 (1.22, 3.47)*	2.71 (2.23, 3.29)*
Region				
Tigray			1	1
Afar			2.07 (1.01, 4.27)*	1.32 (0.61, 2.86)
Amhara			3.72 (1.86, 7.43)*	3.40 (1.60, 7.19)*
Oromia			4.86 (2.53, 9.31)*	3.72 (1.84, 7.52)*
Somali			4.92 (2.57, 9.40)*	3.36 (1.66, 6.80)*
Benishangul			3.34 (1.61, 6.94)*	2.58 (1.19, 5.60)*
SNNPR			2.92 (1.46, 5.84)*	2.62 (1.25, 5.49)*
Gambela			3.16 (1.48, 6.76)*	1.95 (0.86, 4.43)
Harari			3.28 (1.48, 7.26)*	3.53 (1.50, 8.32)*
Addis Ababa			0.40 (0.05, 3.29)	0.49 (0.06, 4.01)
Dire Dawa			3.01 (1.24, 7.34)*	3.11 (1.20, 8.03)*
Source of drinking water				
Protected			1	1
Unprotected			1.26 (1.05, 1.53)*	1.35 (1.16, 1.58)*
Improved toilet facility				
Yes			1	1
No			1.43 (1.09, 1.88)*	1.78 (1.50, 2.12)*

1 Reference category for categorical variable and * reference *p*-value < 0.001

### Individual-level factors

The odds of neonatal death among multiple birth children were 3.18 (AOR = 3.18; 95% CI 2.78, 3.63) times higher as compared to singletons. The odds of neonatal death who are born at home were 1.99 (AOR = 1.99; 95% CI 1.48, 2.69) times higher as compared to children who are born at the health facility. The odds of neonatal death among preterm was 5.97 (AOR = 5.97; 95% CI 4.96, 7.20) times higher compared to term birth. The odds of neonatal death among mothers who had no antenatal care service visit during their pregnancy was 2.33 (AOR = 2.33; 95% CI 2.09, 2.61) times higher as compared to women who had ≥ 4 ANC visits. The odds of neonatal death among mothers who did not receive TT injection during pregnancy was 2.28 (AOR = 2.28; 95% CI 1.92, 2.71) times higher as compared to mothers who received TT injections 4 and above times. The odds of neonatal death among mothers of age group 35–49 were 2.03 (AOR = 2.03; 95% CI 1.68, 2.46) times higher as compared to mothers age group 15–24 respectively. The odds of neonatal death among neonates whose father had no education had 1.32 (AOR = 1.32; 95% CI 1.12, 1.54) times higher than neonates whose father attained secondary education and above. Neonates born to mother who did not have formal education had 1.58 (AOR = 1.58; 95% CI; 1.46, 1.71) times higher likelihood of neonatal death than neonates whose mother who attained secondary education and above. Neonates born less than 24 months of the preceding birth interval were 1.51 (AOR = 1.51; 95% CI 1.35,1.68) times higher odds of neonatal death than children born greater than 23 months of preceding birth interval (Table 3).

### Community-level factors

The risk of neonatal death among rural residents was 2.71 (AOR = 2.71; 95% CI 2.23, 3.29) times higher when compared to urban residents. Neonates in Amhara (AOR =3.40; 95% CI 1.60, 7.19), Oromia (AOR 3.72; 95% CI 1.84, 7.52), Somali (AOR =3.36; 95% CI 1.66, 6.80), Dire Dawa (AOR = 3.11; 95% CI 1.20, 8.03), and Harari (AOR = 3.53; 95% CI 1.50, 8.32) regions were more likely to die compared to neonates in Tigray. The odds of neonatal death among households who used unprotected sources of drinking water were 1.35 (AOR = 1.35; 95% CI 1.16, 1.58) times higher when compared to households who a used protected source of drinking water. The mortality risk of neonates among households that did not have an improved toilet facility was 1.78 (AOR = 1.78; 95% CI 1.50, 2.12) times higher when compared to households who have improved toilet facility (Table 3).

### Measures of variation (random effects)

Table 4 provides the findings of the random effects model. The rate of neonatal mortality varied among clusters (communities). In other words, the neonatal mortality rate has not been spread uniformly across clusters (communities). A significant variance of infant mortality at the community level was seen in the result of the null model (model 1). The finding shows that 38.18% was correlated with infant mortality at the community level. There is a significant variation of infant mortality across clusters (communities) after the inclusion of both the individual- and community-level variables in the model (model IV). In model IV, approximately 85.6% of the variation in the risk of neonatal death was accounted for by the individual- and community-level factors. The MOR for neonatal death was 3.87 in the null model which revealed that there was variation between communities (clustering) (3.87 times higher than the reference (MOR = 1)). When both individual and community variables were applied to the model, the unexplained community variance of neonatal death decreased to a MOR of 1.67. This showed that when considering both individual and community variables, the effects of clustering are still statistically significant (Table 4).

**Table 4:** Measure of variation on individual and community level risk factors of neonatal in rural Ethiopia, EDHS 2016 dataset

Measure of variation	Model I (null model)	Model II	Model III	Model IV (full model)
Variance (SE)	2.03 (0.45)*	0.54 (0.24)*	0.87 (0.28)*	0.29 (0.21)*
PCV (%)	Reference	73.65	57.09	85.57
ICC (%)	38.16	13.99	20.93	8.18
MOR	3.87	2.00	2.43	1.67
Model fit statistics				
DIC (-2log likelihood)	2026.70	1670.11	1922.64	1646.20
AIC	2030.70	1724.11	1952.65	1694.20
BIC	2042.31	1880.81	2039.70	1833.48

*Reference *p*-value < 0.001

## Discussion

The objective of this study is to assess the key determinants of neonatal mortality in Ethiopia. A total of 2449 neonates nested with 443 clusters were included from the 2016 EDHS data. In Ethiopia, the neonatal mortality rate in 2016 was 29 deaths per 1000 live births [9]. This death rate is higher than 19.6 deaths per 1000 live births in Kenya [26], and 10.7 deaths per 1000 live births in South Africa [26]. This could be due to the various health policies implemented in the countries as well as different levels of economic status. The random effects model results showed that both individual- and community-level factors explained about 85.57% of the variance observed for neonatal death. A similar finding was also found in Ethiopia [27].

This study showed that ANC+ visit was a significant factor in neonatal death. When the ANC visit increases, the risk of neonatal death was significantly decreased. The result of this study is in line with other studies [11, 13, 15]. The possible explanation for this result may be that the ANC visit is necessary to improve the health of mothers and fetuses by reducing the complication of pregnancy. Place of delivery was a significant predictor of neonatal mortality. Neonates born at institutional health facilities have a lower risk of death compared to neonates who were born at home. This finding was in agreement with a study done in Ethiopia [13] and Tanzania [28]. As compared to the short birth intervals, long birth intervals were lower risk of infant death, and the risk of neonatal death decrease as the previous birth interval increased. In mothers with short birth interval, the risk of obstetric complication is higher than in those with long birth intervals [29]. A study from Cambodia [23] and Nigeria [30] consistently reported that a long birth interval reduces the risk of neonatal death. Birth order also had a significant influence on neonatal mortality in Ethiopia. The risk of neonatal mortality increased with an increase in the neonatal birth order. This is anticipated that the amount of child care decreases as birth order increases as the mother have more children to care for. This result supported what prior studies have reported [28, 31].

Wealth index was among the significant factors associated with neonatal death. The rich household wealth index was associated with a lower incidence of experiencing neonatal mortality. The possible response might be due to poor nutrition and difficulty accessing health services. Neonates born from high-income households would be able to meet needs and services such as health facilities, quality of life, quality of water, and increased provision of sanitation [32]. These results were consistent with previous reports [18, 31], which suggest that a lower wealth quintile was associated with child mortality. Mothers who received TT injection during pregnancy were less likely to lose their babies during the neonatal period compared to mothers who did not receive any TT injection during pregnancy. This result is consistent with [18]. This may be because TT injection is successful in producing protective antibodies against neonatal tetanus.

Compared to neonates who were never breastfed, neonates who were breastfed from their mother had a lower risk of death. The potential explanation for this may be that breastfeeding protects the babies from infectious diseases because the liquid of the breast is rich in antibodies and white cells. This result is in agreement with previous findings [13–15].

Neonatal sex was significantly correlated with neonatal mortality. The risk of neonatal mortality in females was lower than in male births. This is because early fetal lung maturity is more likely to occur in a female neonate, which will protect against respiratory diseases [33]. This finding is in line with findings from other studies [31, 34]. The birth type was a statistically significant predictor of neonatal mortality. Among multiple births, the risk of neonatal death was higher than singleton births. Due to food intake, multiple births have a lower weight competition [24]. This result is similar to [19]. Besides, the risks of death for neonates with larger birth size were higher than average birth size. This result is similar to [19, 34].

Compared to neonates whose parents did not attend formal education, neonates whose parents attended formal education had a lower risk of neonatal death. The reason behind this is educated parents tended to know about their child’s health status at an earlier stage and begin treatment due to this their baby had a lower risk of death. In addition, educated parents are found to take better care of the neonates during antenatal and postnatal times [35]. This result is similar to previous studies [19, 31]. The age of the mother was an important predictor of neonatal mortality. The older mother had a higher experiencing neonatal mortality than that of the younger mother. This result is in line with the previous findings in Tanzania [28]. In addition, the high risk of experiencing neonatal mortality was correlated with early age at first births (less than 17 years). Previous studies have also shown that mothers who were first born at an early age appear to have neonatal mortality [31].

The findings also showed that place of residence was a significant risk factor for neonatal mortality. Compared to neonates born in rural areas, the risk of neonatal death among neonates born in rural areas was higher. This is because in urban areas newborns have more access to health care and all other critical health-related facilities that are necessary for newborn survival. These studies also agree with the previous study [18, 27]. Furthermore, geographical regions were statistically associated with neonatal death. Mothers from the regions of Amhara, Oromia, Somali, Dire Dawa, and Harari had a higher risk of experiencing neonatal death as compared to in the Tigray region. The possible reason for this regional variation is that the implementation of good health policies differs between regions. This is similar to the previous study [27].

The finding also indicated that the source of drinking water was found to be the most important significant predictor of neonatal mortality. The risk of neonatal death was higher among households using a non-protected source of drinking water than among those households using a protected source of drinking water. Protected sources of drinking water supply are less likely to be infected and are less likely to prevent water-related diseases such as infections and cholera. This finding is in line with [16, 17]. Compared to households that have improved toilet facilities, the mortality risk of neonates among household that has not improved toilet facilities was higher. Access to modern sanitation services such as a flush toilet has decreased the incidence of diarrhea and consequently reduced neonatal death. It is supported by other findings in Nepal [18] and Bangladesh [19].

### Strengths and limitations of the study

This study used 2016 EDHS data with large sample size and high-quality data which reduced the risk of sampling bias and measurement bias. In its design, confounding was controlled for through the proper formation of subcategories of the predictor variables and covariates. This study also uses multilevel analysis to avoid the cluster effect. It is difficult to measure the causal effects, and it is not possible to know whether the data are time dependent or not.

## Conclusion

The death of a newborn is still a public health problem in Ethiopia. This study attempted to identify the key risk factor of and assessing the cluster variation of neonatal death in the country. The individual-level factors associated with neonatal mortality were the sex of the neonates, age at first birth, number of ANC visits, preceding birth interval, birth order, number of TT injections during pregnancy, type of birth, place of delivery, size of neonates at birth, duration of breastfeeding, duration of pregnancy, mothers age, husbands’ educational status, maternal educational status, and wealth index, while place of residence, region, source of drinking water, and type of toilet facility were important factors associated with neonatal mortality at the community level. The findings suggest that attention be paid to education-based programs for mothers that would highlight the benefits of delivery care services, such as ANC visits, TT injections, and facility births. Meanwhile, public health initiatives should focus on expanding access to quality sanitation facilities, especially for latrines and drinking water that could improve neonatal health at the community-level as a whole.

## Data Availability

The data is available and may be delivered upon request.

## Abbreviations

AIC: Akaike’s information criterion
ANC: Antenatal care
AOR: Adjusted odds ratio
CI: Confidence intervals
CSA: Central Statistical Agency
DIC: Deviance information criterion
EAs: Enumeration areas
EDHS: Ethiopian Demographic and Health Survey
EPHI: Ethiopian Health Institute
FMoH: Federal Ministry of Health
ICC: Intracluster correlation
LRT: Likelihood ratio test
MOR: Median odds ratio
PCV: Proportional change in variance
SNNPR: Southern Nations, Nationalities, and People Region
TT: Tetanus toxoid
